# Supplementary figures and images for: Between-subject correlation of heart rate variability predicts movie preferences
Source: PLoS One. 2021 Feb 24;16(2):e0247625. doi: 10.1371/journal.pone.0247625 (PMC7904173; doi:10.1371/journal.pone.0247625)

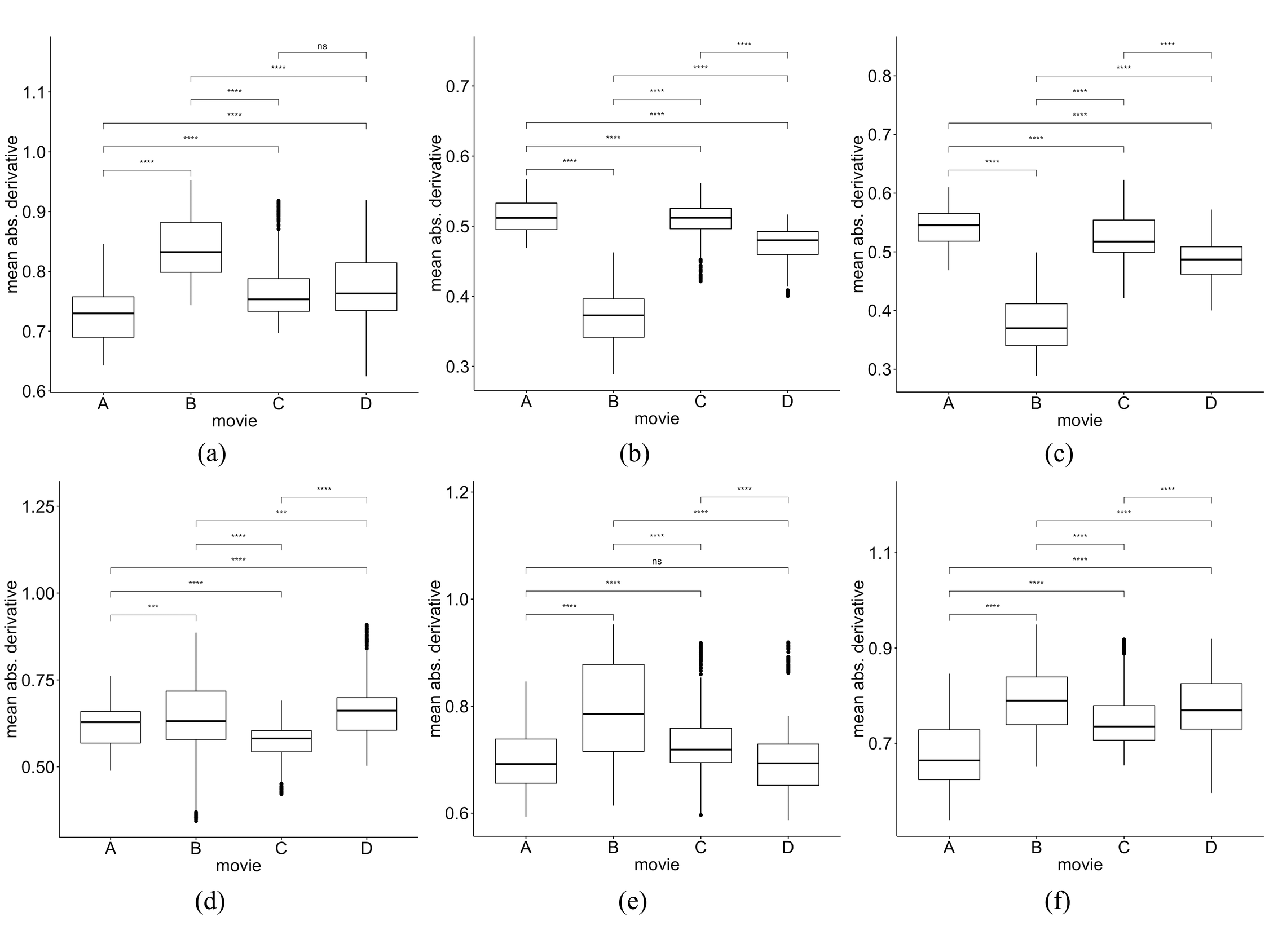

Supplement: S1 Fig — Boxplots of the mean absolute derivatives from PPI diagrams of each movie grouped by video cuts: (a) the “most aroused” cut; (b) the “least aroused” cut; (c) the “most synchronous” cut; (d) the random cut; (e) the female “most aroused” cut; (f) the male “most aroused” cut. (TIF) [file pone.0247625.s001.tif]

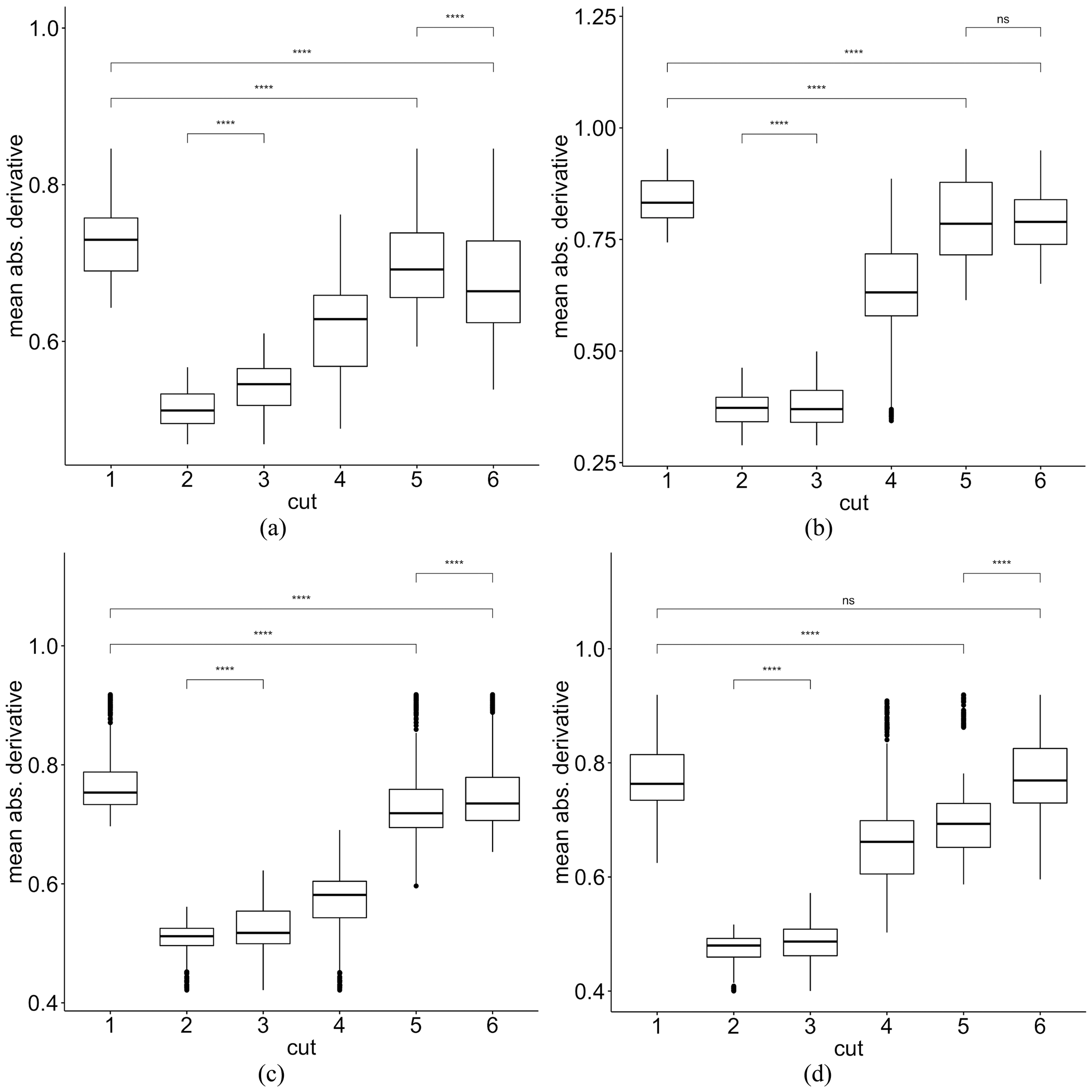

Supplement: S2 Fig — Boxplots of the mean absolute derivatives from PPI diagrams of each cut grouped by movies: (a) Thai commercials; (b) Roma; (c) 2001: A Space Odyssey; (d) Mission Impossible: Rogue Nation. (TIF) [file pone.0247625.s002.tif]
