# Supplementary material for: Between-subject correlation of heart rate variability predicts movie preferences
Source: PLoS One. 2021 Feb 24;16(2):e0247625. doi: 10.1371/journal.pone.0247625 (PMC7904173; doi:10.1371/journal.pone.0247625)
Supplement: S1 Table — Note. * p < .05, ** p < .01, *** p < .001, **** p < .0001. (DOCX) [file pone.0247625.s003.docx]

**S1 Table. Chi-Square Goodness of Fit Test for Comparison 2A (proportions in parentheses).**

|  | **Thai commercials** | **Roma** | **2001: A Space Odyssey** | **Mission Impossible: Rogue Nation** | **Total** |
| --- | --- | --- | --- | --- | --- |
| **most aroused** | 4 | 6 | 6 | 4 | 20 (0.833) |
| **random** | 2 | 0 | 0 | 2 | 4 (0.167) |
| **χ^2^** | - | - | - | - | 10.7 ** |
| **p-value** | - | - | - | - | 0.001 |

*Note. * p<.05, ** p<.01, *** p<.001, **** p<.0001*
