# Supplementary material for: Between-subject correlation of heart rate variability predicts movie preferences
Source: PLoS One. 2021 Feb 24;16(2):e0247625. doi: 10.1371/journal.pone.0247625 (PMC7904173; doi:10.1371/journal.pone.0247625)
Supplement: S3 Table — Note. * p < .05, ** p < .01, *** p < .001, **** p < .0001. (DOCX) [file pone.0247625.s005.docx]

**S3 Table. Chi-Square Goodness of Fit Test for Comparison 3A.**

|  | **Roma** | **2001: A Space Odyssey** | **Mission Impossible: Rogue Nation** | **Total** |
| --- | --- | --- | --- | --- |
| **most aroused** | 12 (0.750) | 12 (0.750) | 8 (0.444) | 32 (0.640) |
| **random** | 4 (0.250) | 4 (0.250) | 10 (0.556) | 18 (0.360) |
| **χ^2^** | 4.00 * | 4.00 * | 0.222 | 3.92 * |
| **p-value** | 0.046 | 0.046 | 0.637 | 0.048 |

*Note. * p<.05, ** p<.01, *** p<.001, **** p<.0001*
