# Supplementary material for: Between-subject correlation of heart rate variability predicts movie preferences
Source: PLoS One. 2021 Feb 24;16(2):e0247625. doi: 10.1371/journal.pone.0247625 (PMC7904173; doi:10.1371/journal.pone.0247625)
Supplement: S4 Table — Note. * p < .05, ** p < .01, *** p < .001, **** p < .0001. (DOCX) [file pone.0247625.s006.docx]

**S4 Table. Chi-Square Goodness of Fit Test for Comparison 3B.**

|  | **Roma** | **2001: A Space Odyssey** | **Mission Impossible: Rogue Nation** | **Total** |
| --- | --- | --- | --- | --- |
| **most aroused** | 6 (0.286) | 8 (0.444) | 6 (0.545) | 20 (0.400) |
| **least aroused** | 15 (0.714) | 10 (0.556) | 5 (0.455) | 30 (0.600) |
| **χ^2^** | 3.86 * | 0.222 | 0.0909 | 2.00 |
| **p-value** | 0.050 | 0.637 | 0.763 | 0.157 |

*Note. * p<.05, ** p<.01, *** p<.001, **** p<.0001*
