# Supplementary material for: Between-subject correlation of heart rate variability predicts movie preferences
Source: PLoS One. 2021 Feb 24;16(2):e0247625. doi: 10.1371/journal.pone.0247625 (PMC7904173; doi:10.1371/journal.pone.0247625)
Supplement: S5 Table — Note. * p < .05, ** p < .01, *** p < .001, **** p < .0001. (DOCX) [file pone.0247625.s007.docx]

**S5 Table. Chi-Square Goodness of Fit Test for Comparison 3C.**

|  | **Roma** | **2001: A Space Odyssey** | **Mission Impossible: Rogue Nation** | **Total** |
| --- | --- | --- | --- | --- |
| **least aroused** | 13 (0.765) | 12 (0.632) | 6 (0.429) | 31 (0.620) |
| **random** | 4 (0.235) | 7 (0.368) | 8 (0.561) | 19 (0.380) |
| **χ^2^** | 4.76 * | 1.32 | 0.286 | 2.88 |
| **p-value** | 0.029 | 0.251 | 0.593 | 0.090 |

*Note. * p<.05, ** p<.01, *** p<.001, **** p<.0001*
