# Supplementary material for: Between-subject correlation of heart rate variability predicts movie preferences
Source: PLoS One. 2021 Feb 24;16(2):e0247625. doi: 10.1371/journal.pone.0247625 (PMC7904173; doi:10.1371/journal.pone.0247625)
Supplement: S6 Table — Note. * p < .05, ** p < .01, *** p < .001, **** p < .0001. (DOCX) [file pone.0247625.s008.docx]

**S6 Table. Chi-Square Goodness of Fit Test for Comparison 3D.**

|  | **Roma** | **2001: A Space Odyssey** | **Mission Impossible: Rogue Nation** | **Total** |
| --- | --- | --- | --- | --- |
| **most synchronous** | 11 (0.733) | 17 (0.850) | 4 (0.267) | 32 (0.640) |
| **random** | 4 (0.267) | 3 (0.150) | 11 (0.733) | 18 (0.360) |
| **χ^2^** | 3.27 | 9.80 ** | 3.27 | 3.92 * |
| **p-value** | 0.071 | 0.002 | 0.071 | 0.048 |

*Note. * p<.05, ** p<.01, *** p<.001, **** p<.0001*
