# Supplementary material for: Between-subject correlation of heart rate variability predicts movie preferences
Source: PLoS One. 2021 Feb 24;16(2):e0247625. doi: 10.1371/journal.pone.0247625 (PMC7904173; doi:10.1371/journal.pone.0247625)
Supplement: S7 Table — Note. * p < .05, ** p < .01, *** p < .001, **** p < .0001. (DOCX) [file pone.0247625.s009.docx]

**S7 Table. Chi-Square Goodness of Fit Test for Comparisons 3A, 3C, and 3D.**

|  | **Roma** | **2001: A Space Odyssey** | **Mission Impossible: Rogue Nation** | **Total** |
| --- | --- | --- | --- | --- |
| **HRV based** | 36 (0.750) | 41 (0.745) | 18 (0.383) | 95 (0.633) |
| **random** | 12 (0.250) | 14 (0.255) | 29 (0.617) | 55 (0.367) |
| **χ^2^** | 12.0 *** | 13.3 *** | 2.57 | 10.7 ** |
| **p-value** | < .001 | < .001 | 0.109 | 0.001 |

*Note. * p<.05, ** p<.01, *** p<.001, **** p<.0001*
