# Supplementary material for: Between-subject correlation of heart rate variability predicts movie preferences
Source: PLoS One. 2021 Feb 24;16(2):e0247625. doi: 10.1371/journal.pone.0247625 (PMC7904173; doi:10.1371/journal.pone.0247625)
Supplement: S8 Table — Note. * p < .05, ** p < .01, *** p < .001, **** p < .0001. (DOCX) [file pone.0247625.s010.docx]

**S8 Table. Chi-Square Goodness of Fit Test for Comparison 3E by movie.**

|  | **Roma** | **2001: A Space Odyssey** | **Mission Impossible: Rogue Nation** | **Total** |
| --- | --- | --- | --- | --- |
| **female most aroused** | 8 (0.258) | 23 (0.622) | 8 (0.250) | 39 (0.390) |
| **male most aroused** | 23 (0.742) | 14 (0.378) | 24 (0.750) | 61 (0.610) |
| **χ^2^** | 7.26 ** | 2.19 | 8.00 ** | 4.84 * |
| **p-value** | 0.007 | 0.139 | 0.005 | 0.028 |

*Note. * p<.05, ** p<.01, *** p<.001, **** p<.0001*
