# Supplementary material for: Between-subject correlation of heart rate variability predicts movie preferences
Source: PLoS One. 2021 Feb 24;16(2):e0247625. doi: 10.1371/journal.pone.0247625 (PMC7904173; doi:10.1371/journal.pone.0247625)
Supplement: S9 Table — Note. * p < .05, ** p < .01, *** p < .001, **** p < .0001. (DOCX) [file pone.0247625.s011.docx]

**S9 Table. Chi-Square Goodness of Fit Test for Comparison 3E by gender.**

|  | **female participants** | **male participants** | **Total** |
| --- | --- | --- | --- |
| **female most aroused** | 19 (0.380) | 20 (0.400) | 39 (0.390) |
| **male most aroused** | 31 (0.620) | 30 (0.600) | 61 (0.610) |
| **χ^2^** | 2.88 | 2.00 | 4.84 * |
| **p-value** | 0.090 | 0.157 | 0.028 |

*Note. * p<.05, ** p<.01, *** p<.001, **** p<.0001*
