# Supplementary material for: Between-subject correlation of heart rate variability predicts movie preferences
Source: PLoS One. 2021 Feb 24;16(2):e0247625. doi: 10.1371/journal.pone.0247625 (PMC7904173; doi:10.1371/journal.pone.0247625)
Supplement: S10 Table — Note. * p < .05, ** p < .01, *** p < .001, **** p < .0001. (DOCX) [file pone.0247625.s012.docx]

**S10 Table. Strength-of-preference grouped by movie.**

|  | **movie** | **N** | **Mean** | **SD** |
| --- | --- | --- | --- | --- |
| **strength-of-preference** | Roma | 100 | 0.570 | 0.498 |
|  | 2001: A Space Odyssey | 110 | 0.400 | 0.492 |
|  | Mission Impossible: Rogue Nation | 90 | 0.589 | 0.495 |

*One-Way ANOVA: F=4.59 *, df1=2, df2=297, p=.011*

*Note. * p<.05, ** p<.01, *** p<.001, **** p<.0001*
