# Supplementary material for: Between-subject correlation of heart rate variability predicts movie preferences
Source: PLoS One. 2021 Feb 24;16(2):e0247625. doi: 10.1371/journal.pone.0247625 (PMC7904173; doi:10.1371/journal.pone.0247625)
Supplement: S11 Table — Note. * p < .05, ** p < .01, *** p < .001, **** p < .0001. (DOCX) [file pone.0247625.s013.docx]

**S11 Table. Tukey Post-Hoc Tests on Strength-of-preference grouped by movie.**

|  |  | **Roma** | **2001: A Space Odyssey** | **Mission Impossible: Rogue Nation** |
| --- | --- | --- | --- | --- |
| **Roma** | Mean difference  p-value | - | 0.170 *  0.036 | -0.0189  0.963 |
| **2001: A Space Odyssey** | Mean difference  p-value | - | - | -0.1889 *  0.021 |
| **Mission Impossible: Rogue Nation** | Mean difference  p-value | - | - | - |

*Note. * p<.05, ** p<.01, *** p<.001, **** p<.0001*
