# Supplementary material for: Between-subject correlation of heart rate variability predicts movie preferences
Source: PLoS One. 2021 Feb 24;16(2):e0247625. doi: 10.1371/journal.pone.0247625 (PMC7904173; doi:10.1371/journal.pone.0247625)
Supplement: S12 Table — Note. * p < .05, ** p < .01, *** p < .001, **** p < .0001. (DOCX) [file pone.0247625.s014.docx]

**S12 Table. Strength-of-preference grouped by comparison condition.**

|  | **comparison** | **N** | **Mean** | **SD** |
| --- | --- | --- | --- | --- |
| **strength-of-preference** | most aroused vs. least aroused | 50 | 0.440 | 0.501 |
|  | most aroused vs. random | 50 | 0.600 | 0.495 |
|  | least aroused vs. random | 50 | 0.740 | 0.443 |
|  | most synchronous vs. random | 50 | 0.480 | 0.505 |
|  | female most aroused vs. male most aroused | 100 | 0.410 | 0.494 |

*One-Way ANOVA: F=4.53 **, df1=4, df2=295, p=.001*

*Note. * p<.05, ** p<.01, *** p<.001, **** p<.0001*
