# Supplementary material for: Between-subject correlation of heart rate variability predicts movie preferences
Source: PLoS One. 2021 Feb 24;16(2):e0247625. doi: 10.1371/journal.pone.0247625 (PMC7904173; doi:10.1371/journal.pone.0247625)
Supplement: S13 Table — Note. * p < .05, ** p < .01, *** p < .001, **** p < .0001. (DOCX) [file pone.0247625.s015.docx]

**S13 Table. Tukey Post-Hoc Tests on Strength-of-preference grouped by comparison.**

|  |  | **Most aroused vs. least aroused** | **most aroused vs. random** | **least aroused vs. random** | **most synchronous vs. random** | **female most aroused vs. male most aroused** |
| --- | --- | --- | --- | --- | --- | --- |
| **most aroused vs. least aroused** | Mean difference  p-value | - | -0.160  0.476 | -0.300 *  0.020 | -0.0400  0.994 | 0.0300  0.997 |
| **most aroused vs. random** | Mean difference  p-value | - | - | -0.140  0.608 | 0.1200  0.736 | 0.1900  0.167 |
| **least aroused vs. random** | Mean difference  p-value | - | - | - | 0.2600  0.063 | 0.3300 **  0.001 |
| **most synchronous vs. random** | Mean difference  p-value | - | - | - | - | 0.0700  0.922 |
| **female most aroused vs. male most aroused** | Mean difference  p-value | - | - | - | - | - |

*Note. * p<.05, ** p<.01, *** p<.001, **** p<.0001*
