# Supplementary material for: Between-subject correlation of heart rate variability predicts movie preferences
Source: PLoS One. 2021 Feb 24;16(2):e0247625. doi: 10.1371/journal.pone.0247625 (PMC7904173; doi:10.1371/journal.pone.0247625)
Supplement: S2 File — (PDF) [file pone.0247625.s017.pdf]

HKU PSY Questionnaire participant no. ___________

Audience Psychology:

Measuring Emotional Response of Audience based on Heart Rate Variability

Part A:

Gender: M / F Age: ___________ Occupation: _________________

Phone: iPhone / Android (pls specify________________________)

Part B:

1. What is your overall rating of the clip (s)
   (1 is the worst while 10 is the best) = ______________________
2. Which plot/part
   a. is the happiest? ____
   b. is the saddest? ____
   c. is the most memorable? ____________________________________
   d. is the most boring? _______________________________________

3. Will you recommend this compilation of CM to the others? Yes / No
4. Have you watched this clip before? Yes / No

Part C:

1. What is your overall rating of the clip (s)
   (1 is the worst while 10 is the best) = _______________________

2. Which plot/part is
   a. the happiest? ____
   b. Is the saddest? ____
   c. is the most memorable? ____________________________________
   d. is the most boring? _______________________________________

3. Will you recommend this movie to the others? Yes / No
4. Have you watched this clip before? Yes / No

Part D:

1. What is your overall rating of the clip (s)
   (1 is the worst while 10 is the best) = ________________________

2. Which plot/part is
a. the happiest? ____
b. the saddest? ____
c. the most memorable? ____
d. the most boring? ____

3. Will you recommend this movie to the others? Yes / No
4. Have you watched this clip before? Yes / No

Part E:

1. What is your overall rating of the clip (s)
   (1 is the worst while 10 is the best) = ________________________

2. Which plot/part is
a. the most exciting? ____
b. the happiest ____
c. the saddest? ____
d. the most memorable? ____
e. the most boring? ____

3. Will you recommend this movie to the others? Yes / No
4. Have you watched this clip before? Yes / No

Thank you for your participation, pls return this questionnaire to the researcher
