# Supplementary material for: Between-subject correlation of heart rate variability predicts movie preferences
Source: PLoS One. 2021 Feb 24;16(2):e0247625. doi: 10.1371/journal.pone.0247625 (PMC7904173; doi:10.1371/journal.pone.0247625)
Supplement: S3 File — (PDF) [file pone.0247625.s018.pdf]

Correlation Matrix

		01	02	03	05	08	09	10	11	12	13	14	16	17	18	19	20	21
01	Pearson's r	—																
	p-value	—																
	N	—																
02	Pearson's r	0.013**	—															
	p-value	0.002	—															
	N	55185	—															
03	Pearson's r	0.068***	0.064***	—														
	p-value	<.001	<.001	—														
	N	55185	55185	—														
05	Pearson's r	0.058***	0.041***	0.048***	—													
	p-value	<.001	<.001	<.001	—													
	N	55185	55185	55185	—													
08	Pearson's r	0.029***	0.045***	0.068***	0.064***	—												
	p-value	<.001	<.001	<.001	<.001	—												
	N	55185	55185	55185	55185	—												
09	Pearson's r	0.063***	0.043***	0.047***	0.100***	0.048***	—											
	p-value	<.001	<.001	<.001	<.001	<.001	—											
	N	55185	55185	55185	55185	55185	—											
10	Pearson's r	0.054***	0.046***	0.057***	0.062***	0.118***	0.080***	—										
	p-value	<.001	<.001	<.001	<.001	<.001	<.001	—										
	N	55185	55185	55185	55185	55185	55185	—										
11	Pearson's r	-0.074	-0.017	-0.062	-0.014	-0.031	-0.108	-0.063	—									
	p-value	1.000	1.000	1.000	0.999	1.000	1.000	1.000	—									
	N	55185	55185	55185	55185	55185	55185	55185	—									
12	Pearson's r	0.088***	0.027***	0.051***	0.060***	0.082***	0.023***	0.045***	-0.029	—								
	p-value	<.001	<.001	<.001	<.001	<.001	<.001	<.001	1.000	—								
	N	55185	55185	55185	55185	55185	55185	55185	55185	—								
13	Pearson's r	0.026***	0.024***	0.012**	0.019***	-0.001	0.006	0.006	0.026***	0.047***	—							
	p-value	<.001	<.001	0.003	<.001	0.577	0.095	0.095	<.001	<.001	—							
	N	55185	55185	55185	55185	55185	55185	55185	55185	55185	—							
14	Pearson's r	0.019***	-0.013	-0.011	-0.007	-0.002	-0.043	-0.033	0.008*	0.010*	0.023***	—						
	p-value	<.001	0.999	0.995	0.942	0.717	1.000	1.000	0.027	0.013	<.001	—						
	N	55185	55185	55185	55185	55185	55185	55185	55185	55185	55185	—						
16	Pearson's r	0.053***	-0.004	0.012**	0.001	-0.029	0.009*	-0.002	-0.007	-0.009	0.001	-0.005	—					
	p-value	<.001	0.815	0.002	0.442	1.000	0.015	0.713	0.956	0.985	0.375	0.880	—					
	N	55185	55185	55185	55185	55185	55185	55185	55185	55185	55185	55185	—					
17	Pearson's r	-0.020	0.005	-0.042	-0.024	-0.042	-0.013	-0.014	0.019***	-0.020	0.027***	0.024***	0.012**	—				
	p-value	1.000	0.116	1.000	1.000	1.000	0.999	0.999	<.001	1.000	<.001	<.001	0.003	—				
	N	55185	55185	55185	55185	55185	55185	55185	55185	55185	55185	55185	55185	—				
18	Pearson's r	-0.037	0.068***	0.042***	0.018***	0.042***	-0.022	0.020***	-0.019	0.014***	0.028***	0.017***	-0.001	0.011**	—			
	p-value	1.000	<.001	<.001	<.001	<.001	1.000	<.001	1.000	<.001	<.001	<.001	0.607	0.005	—			
	N	55185	55185	55185	55185	55185	55185	55185	55185	55185	55185	55185	55185	55185	—			
19	Pearson's r	0.041***	0.049***	0.057***	0.058***	0.051***	0.064***	0.066***	-0.029	0.033***	0.053***	-0.027	-0.012	-0.025	0.060***	—		
	p-value	<.001	<.001	<.001	<.001	<.001	<.001	<.001	1.000	<.001	<.001	1.000	0.997	1.000	<.001	—		
	N	55185	55185	55185	55185	55185	55185	55185	55185	55185	55185	55185	55185	55185	55185	—		
20	Pearson's r	0.051***	0.054***	0.056***	0.070***	0.001	0.054***	0.040***	-0.037	0.038***	0.018***	0.013***	-0.004	-0.001	0.029***	0.046***	—	
	p-value	<.001	<.001	<.001	<.001	0.377	<.001	<.001	1.000	<.001	<.001	<.001	0.819	0.633	<.001	<.001	—	
	N	55185	55185	55185	55185	55185	55185	55185	55185	55185	55185	55185	55185	55185	55185	55185	—	
21	Pearson's r	0.079***	0.013**	0.004	0.036***	0.011**	0.032***	0.011**	-0.058	0.036***	-0.055	0.005	0.013**	0.019***	-0.015	-0.028	0.029***	—
	p-value	<.001	0.001	0.193	<.001	0.005	<.001	0.006	1.000	<.001	1.000	0.134	0.001	<.001	1.000	1.000	<.001	—
	N	55185	55185	55185	55185	55185	55185	55185	55185	55185	55185	55185	55185	55185	55185	55185	55185	—

Note. H_a is positive correlation

Note. * p < .05, ** p < .01, *** p < .001, one-tailed
